# Non-Secretory Gallbladder Paraganglioma: An Incidental Finding on Laparoscopic Cholecystectomy

**DOI:** 10.7759/cureus.106667

**Published:** 2026-04-08

**Authors:** Sahana R Padumane, Tricia Lowrey, Eric Amy

**Affiliations:** 1 General Surgery, Edward Via College of Osteopathic Medicine, Lafayette, USA; 2 Pathology, ReliaPath, Opelousas, USA; 3 General Surgery, Opelousas General Health System, Opelousas, USA

**Keywords:** conventional laparoscopic cholecystectomy, extra-adrenal paraganglioma, gallbladder paraganglioma, incidental histopathological findings, non‑functional paraganglioma

## Abstract

Paraganglioma is a rare form of tumor, with secretory and non-secretory variants. Due to the rare nature of gallbladder paraganglioma (GP), there are no definitive guidelines for management. We present the case of a 35-year-old female patient who presented with intermittent right upper quadrant abdominal pain. Preoperative imaging indicated the presence of gallstones within the gallbladder lumen. Laparoscopic cholecystectomy was performed, with pathology findings consistent with a paraganglioma. The gallbladder is a rare location to find paraganglionic tissue, with its rarity posing diagnostic challenges with a lack of clear differentiating criteria between benign paraganglionic tissue (paraganglionic rests) and benign paraganglioma tumors. Given the rare nature of this condition and the lack of definitive guidelines related to its diagnosis and management, additional documentation and awareness may assist other medical professionals in recognizing and treating paraganglionic tissue found in atypical sites with more clinical confidence.

## Introduction

Paragangliomas are a rare form of neuroendocrine tumor (NET) most commonly found along the sympathetic chain [1]. While secretory variants are typically associated with the sympathetic chain, non-secretory variants are more commonly associated with parasympathetics and typically found along cranial nerves [1]. The gallbladder is innervated by sympathetic nerves through the celiac plexus and by parasympathetic nerves through the hepatic branch of the right vagus nerve [2]. As such, the presumed origin of paraganglionic tissue in the gallbladder would be neural crest cells, which aberrantly migrate along the autonomic nerve fibers to the gallbladder during development. They originate from chromaffin cells and have malignant potential, though they are most commonly benign [1].

A distinction between paraganglioma (non-epithelial) and epithelial NET must be made due to their common confusion. These two diagnoses have similar histological findings, with both typically exhibiting the classic nesting architecture typical of NETs as well as some neuroendocrine markers [3]. Pan-neuroendocrine markers typically shared between the two entities include chromogranin A, synaptophysin, and insulinoma-associated protein 1 (ISNM1). To differentiate between the two entities, specific immunohistochemical markers must be used, such as GATA-3, which is typically found in non-epithelial NETs and is absent in epithelial NETs, and AE1/3, which is typically found in epithelial NETs and absent in non-epithelial NETs [3].

Pancytokeratin is an antibody cocktail of various markers indicative of epithelial origin, including AE1, AE3, and MNF116. As paraganglionic tissue originates from neural crest cells and not epithelial cells, pancytokeratin can be helpful in distinguishing between epithelial and non-epithelial NETs. A negative pancytokeratin stain would support a non-epithelial neuroendocrine origin, typical for paragangliomas and pheochromocytomas [3].

An additional distinction must be made between paraganglionic rests and paraganglioma tumors. Paraganglia may be encountered as incidental microscopic nests of neuroendocrine cells within surgical specimens, representing non-neoplastic paraganglionic rests rather than true tumors [4]. In contrast, paragangliomas are defined as neuroendocrine neoplasms arising from the paraganglionic tissue, typically forming well-circumscribed lesions with nested (“Zellballen”) architecture [1]. However, the distinction between paraganglioma and paraganglionic rest may be challenging, as both share similar morphologic and immunohistochemical features, and definitive histologic criteria are lacking [3].

Due to the rare nature of gallbladder paraganglioma (GP), there are currently only 26 cases of GP published in the English medical literature, with the first reported case published in 1972 [5]. One report additionally mentioned another case of GP found in the archived files of their pathology lab; however, this report was never published and therefore not included in the current count [6]. The majority of these cases were found incidentally, with the vast majority being managed using cholecystectomy. Due to the neuroendocrine origin of paragangliomas, they can be associated with genetic syndromes such as *MEN2* and *SDH* complex mutations [1]. Since paragangliomas can produce catecholamines in a minority of cases, features of catecholamine excess, such as the “classic triad” of profuse sweating, headache, and palpitations, can be associated with secretory variants [1].

Existing literature states that approximately 20% of extra-adrenal paragangliomas are malignant [1]; however, the statistic for paragangliomas of the gallbladder is not established. Due to the lack of established guidelines to treat this diagnosis, previous cases have used the personal and family history of patients to determine if there was adequate clinical suspicion to warrant further investigation. Notably, patients with an *SDHB* mutation have the highest likelihood of a malignant paraganglioma and therefore may especially benefit from further evaluation for metastatic disease [1].

## Case presentation

Our patient was a 35-year-old female at the time of presentation with a past medical history of hypertension, controlled on Losartan, and previous surgical history of cesarean section and tubal ligation. Several months prior to her presentation, she reported a previous history of difficulty swallowing as well as nausea and vomiting. She underwent a barium swallow study and ultrasound of the thyroid to evaluate her dysphagia in December 2024, both of which had no significant findings, with her symptoms resolving after being placed on a proton-pump inhibitor and without surgical intervention. Due to the resolution of her dysphagia symptoms without further intervention, it was presumed to not be related to the paraganglioma.

**Figure 1:**
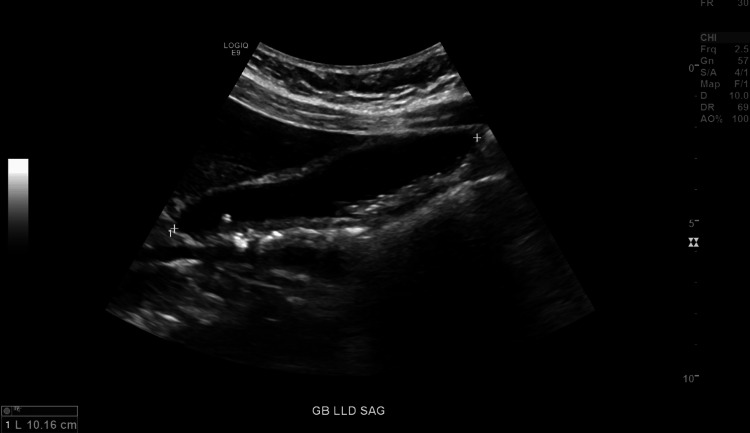
Gallbladder ultrasound with sludge and stone present.

The patient reported having experienced several months of right upper quadrant abdominal pain, consistent with typical symptoms of cholelithiasis, to her primary care provider (PCP). She underwent an ultrasound of the gallbladder in July 2025, which showed mild gallbladder wall thickening, the presence of sludge and stones within the gallbladder lumen, no pericholecystic fluid, a positive sonographic Murphy’s sign, and no obstruction of the bile duct (Figure 1). The confirmation of sludge and stones on imaging confirmed her diagnosis of cholelithiasis and prompted a referral to General Surgery for an elective laparoscopic cholecystectomy. On physical examination, she had non-icteric sclera, with a nondistended abdomen and positive bowel sounds, indicative of a non-emergent state.

**Figure 2:**
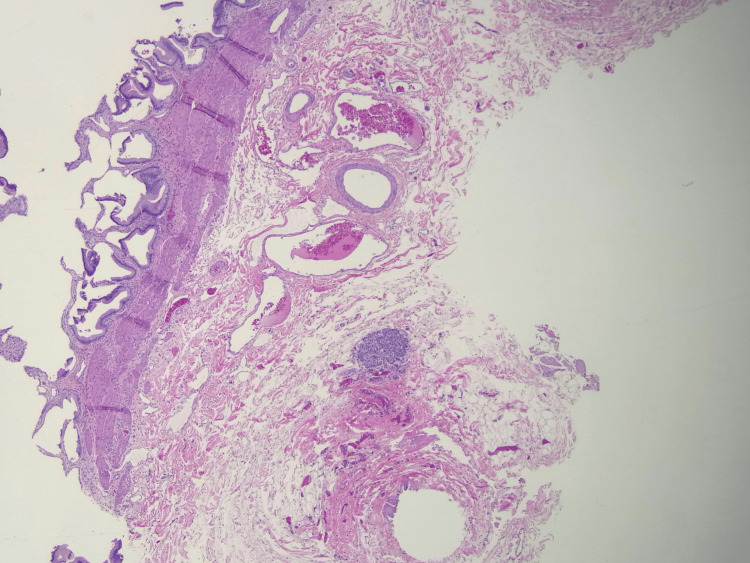
Hematoxylin and eosin section (x2) of the gallbladder wall showing a well-circumscribed submucosal tumor composed of nested cellular aggregates within the muscular and subserosal layers, with intact overlying mucosa.

**Figure 3:**
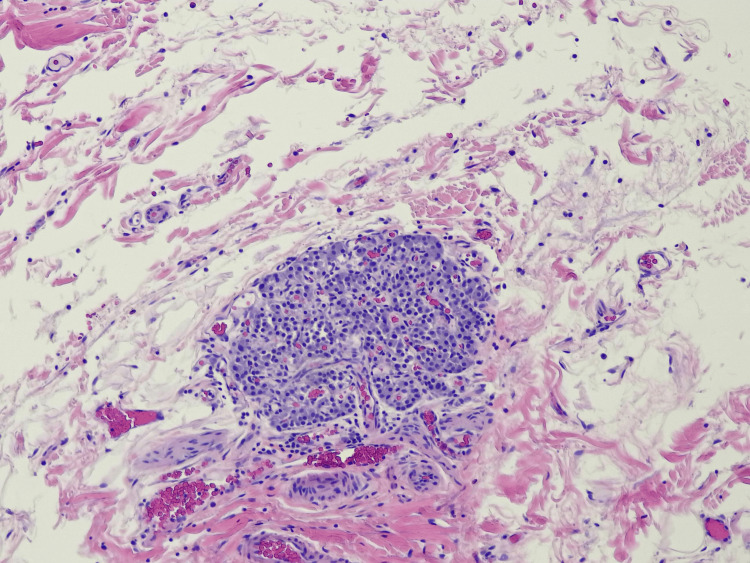
Hematoxylin and eosin view (x10) demonstrating classic Zellballen architecture, with nests of tumor cells separated by delicate fibrovascular stroma and associated small blood vessels.

**Figure 4:**
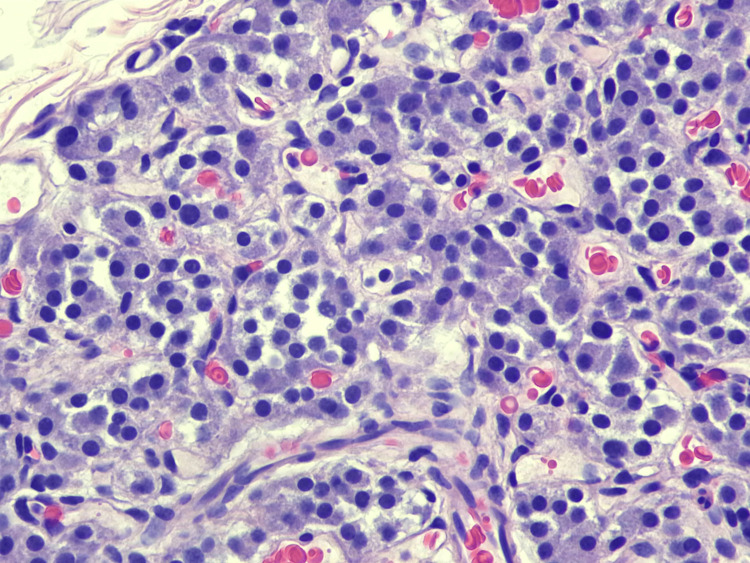
Hematoxylin and eosin view (x40) showing uniform tumor cells with round nuclei, as well as prominent capillary networks, consistent with paraganglionic tissue.

**Figure 5:**
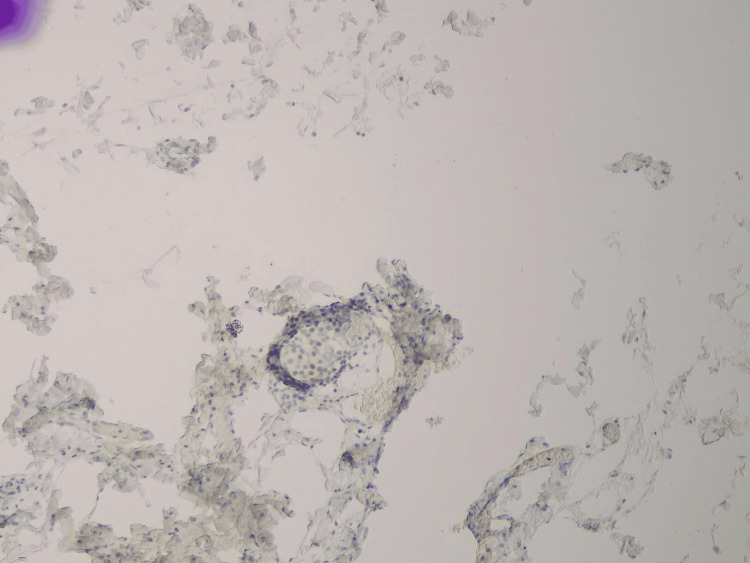
Positive GATA stain (x10) of the paraganglionic tissue.

**Figure 6:**
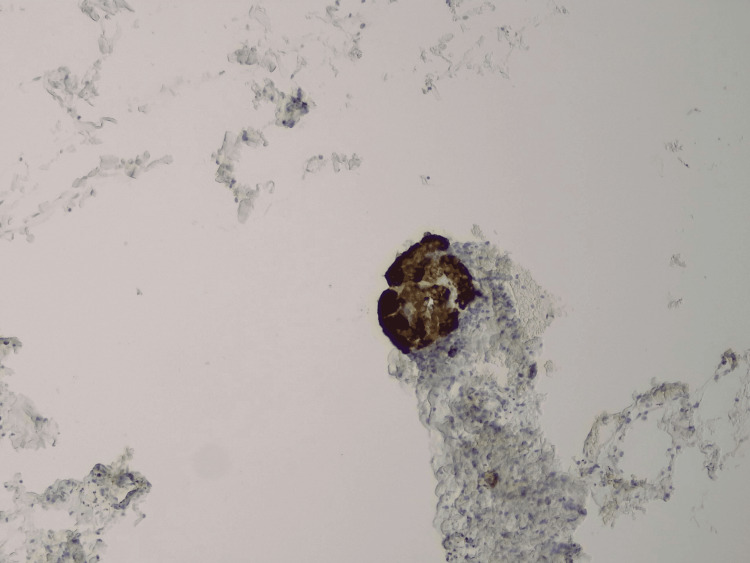
Positive synaptophysin stain (x10) of the paraganglionic tissue.

The surgery was completed without complication. The patient's gallbladder was sent to Pathology, where it was sectioned and examined under the microscope. There were no obvious polyps or masses observed on the mucosal surface. Histologic assessment found amphophilic polygonal cells with round nuclei arranged in a classic Zellballen nesting pattern (Figures 2-4). The cells were negative for pancytokeratin but positive for chromogranin, synaptophysin, GATA-3, and S100 (Figures 5, 6). No mitotic figures were present, and Ki67 was low. The lesion was 0.5 mm and confined to the gallbladder wall with negative margins. These findings were determined by the Pathology group to be consistent with paraganglioma, a rare NET. The diagnosis was made due to its location in the gallbladder wall and a lack of associated neurovascular bundles and/or ganglion cells found on histology, as would typically be found with a paraganglionic rest.

The distinction between paraganglioma and paraganglionic rest in this setting is challenging, particularly given the minute size (0.5 mm) and incidental nature of the finding. Although the lesion demonstrates classic "Zellballen" architecture and immunohistochemical features of paraganglionic differentiation, these findings are not specific for neoplasia. In the absence of a grossly apparent or expansile mass, this lesion may alternatively represent a paraganglionic rest. Due to the lack of clear diagnostic criteria and established differentiating factors between paraganglioma and paraganglionic rests, the designation of paraganglioma in this case should be interpreted with caution.

Paragangliomas have the potential to be of a benign or a malignant nature [1]. Since the patient did not exhibit any symptoms consistent with a secretory paraganglioma, such as hypertensive episodes, and had no elevation of plasma metanephrines, the clinical suspicion for a secretory variant of the paraganglioma was assessed to be low. The patient underwent a contrast CT scan of the abdomen and pelvis to assess for additional tumors approximately one month post-operation. The CT scan showed no remarkable findings, and thus the patient continued regular follow-up with her PCP.

## Discussion

A paraganglioma finding in the gallbladder is an unusual finding due to its rarity. Similar to other GP cases previously reported on in the literature, it does not appear that the tumor was producing any hormones and was discovered incidentally (Table 1). Thus far, there have been no reported GP of the secretory variant [5-27]. However, there have been reports on hemorrhagic GPs that lead to minor complications during removal [9], but our case was a completely incidental finding with no complications or gross abnormalities noted during surgery.

**Table 1:** GB paraganglioma cases reported in the English medical literature since 1972, in chronological order. Asx, asymptomatic; Cg, chromogranin; CT, computed tomography; F, female; GB, gallbladder; HTN, hypertension; IBD, inflammatory bowel disease; M, male; MRI, magnetic resonance imaging; NM, not mentioned; PET, positron emission tomography; PSC, primary sclerosing cholangitis; RUQ, right upper quadrant; NSE, neuron-specific enolase; Syn, synaptophysin

Case	Source	Year published	Age, sex	Clinical presentation	Size (cm)	Management	Staining
1	Miller et al. [5]	1972	57, M	Recurrent hematemesis	3	Cholecystectomy	NM
2	Wolff [6]	1973	32, F	Cholelithiasis	NM	Cholecystectomy	NM
3	Wolff [6]	1973	52, F	Cholelithiasis	NM	Cholecystectomy	NM
4	Wolff [6]	1973	59, F	Cholelithiasis	NM	Cholecystectomy	NM
5	Kawabata . [7]	1999	51, F	Mildly elevated transaminases	NM	Cholecystectomy	CgA, S100
6	Kawabata [7]	1999	55, F	Epigastric pain	NM	Cholecystectomy	CgA, S100
7	Hirano [8]	2000	58, F	Right hypochondrial pain	1.3 × 0.9	Cholecystectomy	NM
8	Cho et al. [9]	2001	45, F	RUQ pain, hemorrhage	2.5	Cholecystectomy	NSE, Cg, Syn, and S100
9	Mehra et al. [10]	2005	36, M	Asx, incidental finding following cholecystectomy done during Roux-en-Y bypass	1.5	Cholecystectomy	Cg A, Syn, S100
10	Koplay et al. [11]	2014	57, M	RUQ pain	2.5	Cholecystectomy	Syn, Vimentin, Cg, S100
11	Oztas et al. [12]	2015	61, F	Suspected GB cancer, former kidney malignancy	2	Cholecystectomy	NM
12	Ece et al. [13]	2015	57, F	RUQ pain	1.8	Cholecystectomy	Vimentin, Syn, S100
13	AlMarzooqi et al. [14]	2018	NM	RUQ pain	0.225	Cholecystectomy	Syn, Cg, SOX10
14	Abdul Sater et al. [15]	2019	36, M	Mild HTN, tinnitus, multifocal SDHD paragangliomas, hyper-enhancing GB lesion on MRI, and avidity on DOTATATE PET	2.1	Cholecystectomy	Syn, Cg, and S100
15	Mahin et al. [16]	2019	79, M	Asx, incidental GB wall mass found on CT of the chest	3 × 3	Cholecystectomy	Syn, Cg, SOX10
16	Corten et al. [17]	2019	27, F	RUQ pain	0.03	Cholecystectomy	NM
17	D'John et al. [18]	2020	63, F	RUQ pain	<1	Cholecystectomy	Syn, Cg, S100
18	Aaquist et al. [19]	2020	74, M	Asx, incidental finding on DOTATATE PET scan	2.2 × 1.6 × 1.1	Cholecystectomy	SDHA and SDHB mutation
19	Song et al. [20]	2021	48, F	Abdominal pain	1.6	Cholecystectomy	Syn, Cg, CD56
20	Shreya et al. [21]	2021	72, F	Bilateral otorrhea, CT and DOTATATE PET, mass in the left hypotympanum, bilateral carotid spaces, GB mass	2.3x 2.2	No intervention	NM
21	Xia et al. [22]	2023	48, F	Liver mass	6.0 × 4.0 × 3.5	Cholecystectomy	Cg A, NSE, Syn, and S100
22	Do et al. [23]	2023	53, M	RUQ pain	0.5	Cholecystectomy	Cg A, Syn, S100
23	Hingway et al. [24]	2024	36, F	RUQ pain	NM	Cholecystectomy	Cg A, Syn, S100
24	Dominkovic et al. [25]	2025	54, F	Chronic RUQ pain, IBD, PSC	0.7 x 0.8	Cholecystectomy	Cg, Syn, S100
25	Lee et al. [26]	2025	50, F	Family history of malignant carotid paraganglioma, SDHB variant	2.5	Cholecystectomy	Cytokeratin AE1/AE3, Syn, and Cg
26	Mawlaalduwilah et al. [27]	2025	37, F	RUQ pain	<0.1	Cholecystectomy	Syn, S100
27	This case	2025	35, F	RUQ pain	0.05	Cholecystectomy	Cg, Syn, GATA-3, and S100

Due to the rare nature of GP tumors, there are currently no definitive guidelines available for the management of this disease. Minimally invasive surgical techniques, such as laparoscopic surgery, are generally regarded as the first-line treatment for paragangliomas of the abdomen [1]. Prognosis of benign paraganglioma is very optimistic after complete resection, with a likelihood of recurrence within 10 years estimated to be less than 10% with no statistical impact on survival [1]. However, recurrence likelihood in patients presenting with metastatic disease is 65% [1].

CT scans have the same sensitivity and specificity for pheochromocytomas and paragangliomas as MRI [28]; therefore, in a patient with no symptoms of catecholamine excess, such as hypertensive episodes, it may be appropriate to evaluate a patient for additional paragangliomas using CT instead of MRI and scheduling regular follow-up. Current literature recommends annual biochemical studies initially to test for excess levels of metanephrines and imaging, with a decrease in frequency over time but lifelong continuation [1]. This is due to the reports in the literature of recurrence occurring even 50 years after initial diagnosis [1]. Therefore, radiographic imaging to evaluate the presence of additional tumors as well as adherence to regular follow-up is imperative.

Due to a low clinical suspicion for hormone secretion in this case and a lack of pertinent family history, the patient underwent a CT scan of the abdomen and pelvis, which was negative, to assess if additional tumors were present. The patient has not yet undergone genetic testing, though genetic testing is recommended for all cases of paraganglioma [1]. Regular follow-ups with the attending surgeon and PCP were recommended. While this case was suspected to be of a benign nature, 31% of paraganglioma cases initially diagnosed as benign may end up recurring [1], though the true statistic for GPs may differ from paragangliomas found in other locations.

## Conclusions

There are no definitive guidelines on the management of GP owing to the limited number of cases of this condition. Similar to most other cases of GP found in the literature, this case of GP was asymptomatic and non-secretory. Characteristics found on staining were similar to other cases as well, with the GP staining positive for chromogranin, synaptophysin, and sustentacular cells staining for S100. GATA-3 also stained positive in this case, confirming our diagnosis of paraganglioma; however, all other cases of GP found in the literature did not mention whether GATA-3 was stained for. Pancytokeratin staining was negative, distinguishing this case from epithelial neuroendocrine pathology. Due to its incidental nature, small size, and lack of clear differentiating criteria, this lesion may instead be interpreted as a paraganglionic rest instead of a paraganglioma tumor.

Much is still unknown about paraganglioma tumors of the gallbladder. We hope that further contributions to this growing body of literature will elucidate unknown aspects of this condition and eventually lead to the establishment of clear diagnostic and treatment guidelines.
